# Supplementary material for: Patterns of Intron Gain and Loss in Fungi
Source: PLoS Biol. 2004 Nov 30;2(12):e422. doi: 10.1371/journal.pbio.0020422 (PMC532390; doi:10.1371/journal.pbio.0020422)
Supplement: Table S1 — Also available at http://genes.mit.edu/NielsenEtAl/. (4.3 MB ZIP). [file pbio.0020422.st001.zip › NielsenEtAl/html/915.html]

AN5162.1.NCU06482.1.MG06371.1.FG05454.1


```
 CLUSTAL W (1.82) Multiple Sequence Alignments - Introns Inserted


Sequence 1: NCU06482.1	417 aa
Sequence 2: MG06371.1	416 aa
Sequence 3: FG05454.1	409 aa
Sequence 4: AN5162.1	405 aa
Alignment Length: 420 aa
Number Identitical Residues: 279 aa
Alignment Score (without introns) 12160


MG06371.1 	MLSRGLRIGRAVQAPLRQRTQAPFVAV----ARRCVTTDAASAHVEKGAVPK0SDDEPFT
NCU06482.1	MFSRAARLSTRVAAPVRARVAAPRVVIPSIAARRSVTTNAASAQLEK-PLPE0SETEPFQ
FG05454.1 	MLSRALRLPR--AVPMRTKLAAPAYTA-----VRSVTTDAASASLSH-SVPK0ADDEPFS
AN5162.1  	MLFRTAWARQ--AAPLRRQAFAP-------LARRSVTTDAASSHAEN--IPE0DENKPFT
          	*: *         .*:* :  **        : *.***:***:  .:  :*:  : :** 

MG06371.1 	VNLSDESFETYELDPPPYTLDVTKKDLKQMYYDMVVVR2QMEMAADRLYKEKKIRGFCHL
NCU06482.1	VTLSDESFETYELDPPPYTLNVTKKELKQMYYDMVVVR2QMEMAADRLYKEKKIRGFCHL
FG05454.1 	VNLSDESFETYELDPPPYTLEVTKKELKDMYREMVVTR2QMEMAADRLYKEKKIRGFCHL
AN5162.1  	VRLSDESFETYEIDPPPYTLEVTKKELKQMYYDMVAMR2RMEMAADRLYKEKKIRGFCHL
          	* **********:*******:****:**:** :**. * :********************

MG06371.1 	STGQEAVAVGIEHAINKSDDVITSYRCHGFAYMRGGTVRSIIGELLGRREGIAYGKGGSM
NCU06482.1	SVGQEAVAVGIEHAIERADDVITSYRCHGFAYMRGGTVRSIIGELLGRRGGIAYGKGGSM
FG05454.1 	STGQEAVAVGIEHAITKEDDIITAYRCHGYALLRGASVRSIIGELLGRREGISYGKGGSM
AN5162.1  	STGQEAVAVGIEHALTREDKIITAYRCHGYAMMRGGTIRSIIGELLGRREGIAYGKGGSM
          	*.************: : *.:**:*****:* :**.::*********** **:*******

MG06371.1 	HMFAKGFYGGNGIVGAQVPVGAGLAFAQKYTGGKKASIILYG~DGASNQGQVFEAFNMAK
NCU06482.1	HMFTKGFYGGNGIVGAQVAVGAGLAFAHKYTGRKNASIILYG1DGASNQGQVFESFNMAK
FG05454.1 	HMFAKGFYGGNGIVGAQVPVGAGLAFAHKYNNNKNASIILYG~DGASNQGQVFEAFNMAK
AN5162.1  	HMFAPNFYGGNGIVGAQVPVGAGLAFAQQYNEEKSTSVVLYG~DGASNQGQVFEAFNMAK
          	***: .************.********::*.  *.:*::*** ***********:*****

MG06371.1 	LWNLPALFGCEN1NKYGMGTSASRSSALTDYYKRGQYIPGLKVNGMDALAVKAAVKYGKE
NCU06482.1	LWNLPALFGCEN1NKYGMGTSAARSSALTDYYKRGQYIPGLKVNGMDVLAVKAAVAYGKQ
FG05454.1 	LWNLPALFGCEN1NKYGMGTAAARSSALTDYYKRGQYIPGLKVNGMDVLAVKAAVKYGKE
AN5162.1  	LWNLPVLFGCEN1NKYGMGTSAARSSALTDYYKRGQYIPGIKVNGMDVLATKAAVKYGKD
          	*****.****** *******:*:*****************:******.**.**** ***:

MG06371.1 	WTESGNGPLVLEYVTYRYGGHSMSDPGTTYRTREEIQRMRSTNDPIAGLKQKMMDWDVVT
NCU06482.1	WTNNDNGPLVLEYVTYRYGGHSMSDPGTTYRTREEIQRMRSTNDPIAGLKQHILEWGVAS
FG05454.1 	YTAADKGPLVLEYVTYRYGGHSMSDPGTTYRTREEIQRMRSTNDPIAGLKQKILDWEITT
AN5162.1  	YAISGNGPLVYEYVTYRYGGHSMSDPGTTYRSREEIQRMRSTQDPIQGLKQKILDWGVMS
          	::  .:**** ********************:**********:*** ****::::* : :

MG06371.1 	EEELKSLDKKARSFVNEEVKAAEAMVPPEPTQQILYEDIYVKGTEPEYIRGRTPDELFYF
NCU06482.1	EEELKNLDKEARAYVNEEVAAAEAMPPPEANQQILFEDIYVKGTEPDFIRGRTPDELFYF
FG05454.1 	EEELKKIDKEARAHVNEEVAAAEAMAAPEAKPEILFEDIYVRGSEPEYIRGRIPEENHYF
AN5162.1  	EEDLKGLDKSARAHVDEEVAIAEKMPLPENNSRILFEDIYVRGSEPRWMRGRTVDETFYY
          	**:** :**.**:.*:***  ** *  ** . .**:*****:*:** ::***  :* .*:

MG06371.1 	ENKN
NCU06482.1	KH--
FG05454.1 	Q---
AN5162.1  	----
          	
```
